# Death of Emperor Frederick III

**Published:** 1888-07

**Authors:** 


### DEATH OF EMPEROR FREDERICK III.

There is a striking parallel between the disease, suffering and death of Emperor Frederick III, of Germany, and the late Ex-President Grant. Both had distinguished themselves as mllitary commanders in the field ; one had but recently relinquished the reins of government over a great nation ; the other had but taken them in hand. In their affliction a heroism superior to that common to battle fields, was evinced and appreciated by the populace with a sympathy so profound as to dwarf all less worthy sentiments, and the medical bulletins became the one engrossing object of enquiry and speculation during the changeful states of the disease to the final end. In both instances the malady in the last stages was conceded to be cancer, and consequently, incurable by any treatment other than the surgeons knife, and absolutely incurable when the free use of the knife upon a vital part would prove fatal. Had the Crown Prince's disorder been pronounced cancer by the eminent physicians in attendance upon him, with a concurrent statement of its incurability, the rule of the empire would have barred him from the throne, but so far as we are advised, the medical gentlemen who had the case in hand were never agreed as to the exact nature of the malady or the best methods of treatment. The home physicians looked with no little jealousy upon the introduction to the case of the accredited physician to the English Queen, an event to which they never became fully reconciled.

From the outset, the advance of Emperor Erederick's disease indicated cancer rather than sarcoma, by its local habit and progress through the tissues. There may have been some political significance, too, in the indecision of the faculty, in view of the rule of succession. However this may be, it is not at all likely that it will ever be made the subject of dispute. All the formalities requisite to succession to the throne were observed save one, which, for prudential reasons, was dispensed with by parliamentary enactment. Nor is it quite established that cancer is an incurable disease. That there have been cases of cure by methods unrecognized by practitioners of the older schools of medicine, is quite well established, and the belief is gaining ground that there is something outside of them yet to be learned in the healing art. But whenever a cure is affected by a specialist it is usual for opposing schools to dispute that the patient was really a cancer subject.

---

PEACH leaves pounded to a pulp and applied to bruise or wound from rusty nail, or a simple cut, will give immediate relief.

